# Dogs display owner-specific expectations based on olfaction

**DOI:** 10.1038/s41598-021-82952-4

**Published:** 2021-02-08

**Authors:** Juliane Bräuer, Damian Blasi

**Affiliations:** 1 Department of Linguistic and Cultural Evolution, Max Planck Institute for the Science of Human History, Kahlaische Strasse 10, 07745 Jena, Germany; 2 Department for Department for General Psychology and Cognitive Neuroscience, Friedrich Schiller University of Jena, Am Steiger 3, 07743 Jena, Germany; 3 Department of Human Evolutionary Biology, Harvard University, Peabody Museum, 5th Floor, 11 Divinity Avenue, Cambridge, MA 02138 USA; 4 Linguistic Convergence Laboratory, National Research University Higher School of Economics, 21/4 Staraya Basmannaya Ulitsa, Building 5, Moscow, Russian Federation

**Keywords:** Psychology, Behavioural ecology

## Abstract

Most current knowledge about dogs’ understanding of, and reacting to, their environment is limited to the visual or auditory modality, but it remains unclear how olfaction and cognition are linked together. Here we investigate how domestic dogs search for their owners using their excellent olfactory sense. We raise the question whether dogs have a representation of someone when they smell their track. The question is what they expect when they follow a trail or whether they perceive an odour as a relevant or non-relevant stimulus. We adopted a classical violation-of-expectation paradigm—and as targets we used two persons that were both important to the dog, usually the owners. In the critical condition subjects could track the odour trail of one target, but at the end of the trail they find another target. Dogs showed an increased activity when the person did not correspond with the trail compared to a control condition. Moreover, we found huge individual differences in searching behaviour supporting the assumption that dogs are only able to smell when they really sniff, and that the temperature has an influence on dogs performance. Results are discussed in the light of how cognitive abilities, motivation and odour perception influence each other.

## Introduction

In the last 30 years there has been a growing scientific interest in dog cognition as dogs have developed special skills to function effectively in the human environment, as a consequence of the oldest domestication process we have evidence for^1^. It is well known that dogs have an excellent olfactory sense, and that they rely heavily on it when exploring the environment or recognizing individuals^2–6^. Dogs can learn to recognize various odours^7,8^, they can be trained to discriminate and indicate the presence of odours of narcotics, explosives, plants, parasites and various diseases such as cancer and diabetes^9–15^. They are also able to match odours^16^, i.e. they can confirm or deny that two odours come from the same source^17,18^.

In spite of these remarkable olfactory skills, an anthropocentric bias in experimental animal cognition resulted in that most current knowledge about dogs’ understanding of, and reacting to, their environment is limited to the visual or auditory modality^19^. Thus, the link between olfaction and cognition remains an important gap in our understanding of the mammalian species most closely associated with humans.

A starting point in this investigation is to determine the relative importance of the olfactory dimension (as opposed to the visual and auditory dimensions) in dogs’ representation of the world. Moreover, the ideal target should be salient and relevant for the dog, ecologically valid, and able to provide multimodal cues for their identification. Those conditions are satisfied by the dog’s owner. Dogs are able to form a special relationship with their owners^20^ and they systematically prefer the human as a social partner in the presence of choice^21^. Likely due to their extended process of domestication, they display complex interactional process with their owners. For example, when dogs are confronted with an unsolvable problem, they look back to their owners to elicit help^22,23^. Similarly, dogs indicate the location of food or toys to their owners when owners have not seen where these rewards were hidden^24–26^. Sometimes they even show the location of objects in which they are not interested in (i.e. a hole-puncher, a vase)—and they do it much more frequently to their owner than to strangers^27,28^. In the Strange Situation Test—a procedure to assess individual differences in attachment behavior by evoking individual's reaction when encountering stress—dogs behave similar to human children^20,29^ and even shelter dogs that were handled by a human for three times for 10 min showed attachment to this person^30^. The overwhelming evidence on the intensity of the dog–human bond has been compared in strength with the mother-infant bond in humans^20,31,32^.

Thus, in the current study we tap on the dogs` motivation to approach their owner to investigate the olfactory component of their representation. In concrete, we focus on the canine ability to locate their owners through their tracks, which occupies a central position in the domesticated animal’s skill repertoire. Previous investigations on the role of olfactory representations entailed by tracking behaviour did not yield conclusive results due to the influence of a number of uncontrolled exogenous factors.

To start with, canine training has a huge effect on tracking behaviour and skill^33^. Trained dogs can determine the direction of an odour trail left by a human an hour ago with as little as five footsteps^34,35^. For untrained dogs the scenario is sharply different: they sometimes seem to be unable to gather odour signals (associated with humans or food) when the target is more than a meter away^36^. Interestingly, dogs often first attempted to solve problems based on the little visual information they have at their disposal rather than on the available olfactory cues. In spite of their ability to successfully collect information through olfaction, untrained family dogs often prioritize other strategies such as a win-stay strategy to solve such tasks^36^.

The reliance on olfactory cues might depend critically on environmental factors. Temperatures above 15 C might reduce the physical capacity of dogs in motion because of hypothermia^37,38^. However, it is not easy to distinguish whether searching dogs show a decrease in detection rates because of their physiological conditions or because of environmental conditions that have impact on the source of the scent (i.e. how it disperses through the air^39^, as air temperature and relative humidity may influence the evaporation rate of the scent source, or the bacterial activity that releases scent vapors^40–42^, see also^43,44^).

Finally, the experimental design should offer a transparent interpretation of behavioural results. Bräuer & Belger (2018) presented dogs with a violation-of-expectation paradigm in which they tracked the odour trail of one target toy (target A), just to find another target toy at the end of the trail (target B)^33^. On the very first trial, dogs showed measurable signs of “surprise” (i.e. further searching for target toy A) presented with target B. However, on subsequent trials dogs did not engage in any further search behaviour, which could be explained by two competing hypotheses. First, dogs did develop an olfactory representation but they were able to pick the smell of previous trials (although the room was cleaned between trials). Second, as dogs were always rewarded with playing as long as they fetched an object, they learned rapidly that it did not matter whether they fetched the toy that corresponded with the odour trail. While all in all the evidence seems to support the notion that they do entertain olfactory representations of the toys, the results are not conclusive.

Hence, in the current study we aimed to investigate how dogs track their owners using the classical violation-of-expectation paradigm of Bräuer & Belger (2018) to answer two questions. Dogs were presented with two persons that were both important to the dog, usually the owners (master and mistress). In the critical condition subjects could track the odour trail of one target (Person A), but at the end of the trail they found another target (Person B). Firstly we explored, whether dogs showed measurable signs of “surprise” and searching (for Person A) when they find Person B, which does not correspond to the odour representation of Person A from the trail. We hypothesized that dogs represent who exactly they smell, when they follow the trail of their owner, i.e. that dogs understand the correspondence between the smell of the owner and the owner him/herself. By testing the dogs only one trial per day with at least a week in between the two trials we excluded that dogs` behavior was influenced by the owners` odour in the trial they experienced before—as in the study of Bräuer & Belger (2018)^33^. Secondly, we analysed how mean temperature of the day and rain influenced the tracking performance of dogs, hypothesizing that dogs perform better when the atmospheric humidity is high and when the mean temperature is low.

## Methods

### Subjects

In total, 54 dogs (28 males and 26 females) of various breeds and ages (ranging from 1–15 years old, mean 6.2 years) participated successfully in this study (see Table 1). All subjects lived with their owners and were registered in our database. Dogs were encouraged to explore all testing rooms prior to the test. They were tested individually, with their owners being the target of their search.

**Table 1:** Subjects included in the study (* passed examination for agility, guard dog, trail dog or hunting dog).

Subject	Breed	Gender	Age
ArH	Mops	Male	8
BeT	Deutsch Langhaar*	Female	4
BiE	Retriever	Male	7
BrH	Schafspudel	Male	4
CaN	Pointer Mix	Male	10
CeB	DSH*	Male	8
ChH	Mix	Male	11
DaU	Labrador	Male	6
DeK	Chihuahua	Male	8
DiW	Lagotto Romagnolo*	Female	6
ElK	Tervueren*	Male	1
ElS	Mix	Female	15
ElT	Deutsch Langhaar*	Female	12
EmH	Golden Retriever	Female	3
EmK	Labrador	Female	10
ErW	Malinois, Mix	Male	7
FaF	Mix	Female	2
FoR	Mix*	Male	10
FrB	Münsterländer*	Female	9
FrM	Berger Picard	Female	8
GeL	Mix, Tervueren	Male	5
GiM	Mix	Male	4
HeJ	Schnauzer	Male	4
HoT	Deutsch Langhaar*	Female	4
HoV	DSH	Male	3
IvS	Nova Scotia Duck Tolling Retri	Male	8
JaH	DSH, Mix, Schnauzer*	Male	4
KaS	Australian Shepard*	Female	7
KiK	Border Collie*	Female	3
KiS	Labrador	Female	2
LaK	Malteser, Mix	Female	7
LaP	Cavalier King Charles Spaniel	Female	5
LiG	Labrador	Female	6
LoD	Labrador, Mix	Male	5
LoW	Labrador, Pudel	Female	3
LoB	Berner Sennenhund, Hovawart	Female	7
LuG	Altdeutscher SH*	Female	3
MaP	Border Collie, Labrador	Female	8
MaR	Landseer	Female	2
MeB	Grosspudel	Male	12
MiR	Mix*	Male	6
MoJ	Flat coated Retriever, Magyar Vizsla, Mix	Female	3
NaB	Border Collie, Mix	Female	4
PaR	Pyrenaenberghund	Male	5
PaB	Mix	Male	9
SeR	Mix	Female	8
ShS	Spitz	Female	6
SkO	Cocker Spaniel	Male	7
TaB	Magyar Vizsla	Male	4
TiH	Mops	Male	7
WiG	Golden Retriever	Male	6
WiM	Mix	Female	5
YoE	Airedale Terrier*	Male	3
YoS	Mix	Male	10

All dog-owner pairs took part voluntarily and owners signed an informed consent form prior to the study. Owners received detailed information about the purpose of the study after the test. The study adhered to the *Guidelines for the use of Animals in Research* and the general guidelines of the Max Planck Society. It was notified to the Official Veterinarian of Jena und Saale-Holzland-Kreis and approved by the Ethic Committee of the Max Planck Society.

### Setup

**Figure 1:**
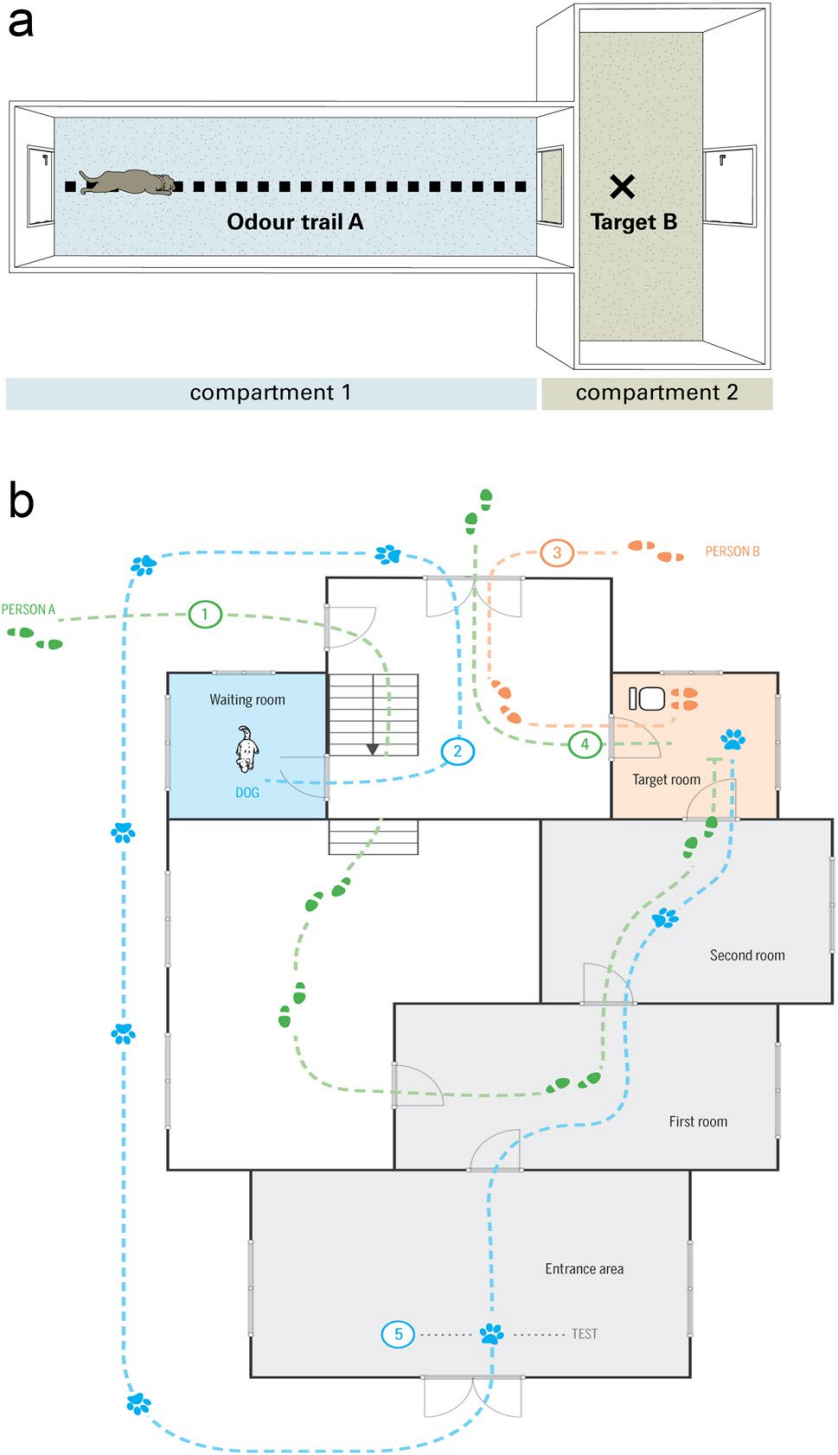
(**a**) General set-up (copyright permission Journal of Comparative Psychology). (**b**) Detailed set-up.

The study was performed in the testing facilities of the dog lab at the Max Planck Institute for the Science of Human History in Jena, see Fig. 1a,b. The main test took place in first room where the search started (13.0 × 5.0 m), a second room (7.0 × 5.0 m) and the target room where the target person sat (3.5 × 3.5 m; this room was a former kitchen that had not been used for 2 years before the experiment started). The shortest distance between the starting point in the first room and the target person in the target room was about 18 m.

The dog entered the first room from an entrance area. All rooms were connected with doors, but the door between entrance and first room was out of Plexiglas and only 1.20 m high.

While the target person walked through the test rooms, dogs stayed in the so-called waiting room (4.0 × 4.0 m) that was isolated from the other test room and the entrance.

Target persons A and B were two persons with a close relationship with the dog—usually the owners (often a couple, but also sometimes one person with their offspring or siblings) or close relatives or friends of the owners. Note that we did not clean the rooms, thus, the odour of the experimenters and other dogs was also present, but we assumed that the smell of the target person was the most important one for the dog subjects. In a few cases more than one dog was tested with the same target person.

### Procedure

For each dog we followed a strict schedule of five steps to guarantee that the dog was not able to perceive the target persons before the test and that the timing of trials was comparable. Target persons were instructed and led by two experimenters E before and after the actual test. For details, see Fig. 1b.

1.) The dog to be tested was brought to the doglab (usually by the owner). The person was not allowed to enter the building. S/he had to hand the dog over at the outer door of the entrance area and had to leave the compound of the institute. The dog stayed in the doglab for about 30 min and had the opportunity to explore the testing area. Then it was brought into the waiting room.

2.) E picked the first target person (A or B) from the main entrance of the institutes` compound and brought her/him directly to the door where she had to enter (see Fig. 1b). S/he was then lead through a stairs down and went through the first room, the second room and entered the target room. While the target person entered the building she was not allowed to speak so that the dog in the waiting room could not hear her/him. E closed all doors behind them and after both had reached the target room, the target person was allowed to speak again. E explained the target person the whole procedure and answered open questions. The target person sat on a chair and was encouraged to read a book or play with her/his mobile.

3.) The dog was brought from the waiting room around the building into the entrance area, here the dog was crossing the scent trail of the first target person once (which was not a problem as this was the trail of the person the dog was following when the test started).

4.) This step depended on the condition.

In the Surprise condition (AB or BA), the second target person (B or A) was picked from another entrance of the institutes` compound and brought directly to another entrance of the building that lead to the target room from the other side. Then target persons were exchanged, the second target person sat on the chair and the first target person left on the same way in which the second target person entered.

In the Control condition (AA) the first target person waited in the target room for about 10 min. S/he then left the room (in the same direction as in the Surprise condition) but immediately entered it again and sat on the chair again. (This was done to make the whole procedure comparable to the Surprise condition).

5.) E opened doors between first room and target room. She then opened the door from the entrance area to the first room to release the dog. Then she immediately left the testing area (heading to the waiting room), so that the dog was alone. Target person (A or B) was strictly forbidden to make any noise and was encouraged to read a book or play with her/his mobile. In case the dog was approaching her/him she was instructed to greet him shortly in a natural way (as if the dog came home from a walk with another person), but not to hold or command him/her. Then she was requested to keep on reading/playing with the mobile.

If the dog found target person in the phase 1, i.e. after 180 s, the trial was over.

If E entered the target room and the dog was not there and had not entered (indicating by a head shake of the target person), E called the name of dog twice and said “Come”—and phase 2 started.

If the dog had not entered after 360 s, the trial was terminated.

### Design

There were two conditions, depending on whether the target person was replaced or not.

In the Control condition (AA) the person was not replaced while in the Surprise condition (AB or BA) was replaced. Each dog was tested one trial per each condition, and one trial per day. Importantly, dogs received two trials—with at least a week in between the two trials so that their searching behavior was not influenced by the odour of their owner in the trial before (see^33^.

Before the test started the two target persons were assigned to be either target person A or target person B. Each trial was identified by the person who generated the trail—indicated by the first letter, and the person who was discovered by the dog—indicated by the second letter. Regarding the order of trials dogs were assigned to one of four orders: AA–AB or AA–BA or AB–AA or BA–AA. Thus, half of the dogs started with a Control condition and half of the dogs started with a Surprise condition; and in the Surprise condition it was varied whether person B generated the trail or was discovered by the dog.

The time delay between the moment when the trial was laid by the owner and the search of the dog was about 15 min.

### Coding and analysis

The main precondition for subjects to be included into the analysis was that they would enter the testing rooms in at least one of the two trials. One dog was excluded for that reason.

Some trials could not or only partly be incorporated into the analysis for the following reasons: dog could participate only one testing day (3 trials); video got lost (2 trials) and target person made a mistake (15 trials). No target person made a noise before dogs approached him/her. Mistakes of the target person included holding the dog, feeding the dog with a treat and commanding him/her. As all this happened in the target room after the approach, the measures approach, latency to approach and the behaviours in the first room could also be included into the analysis in trials in which target persons made a mistake.

To assess inter-observer reliability, one independent and naïve observer scored a randomly selected sample of 20% of the trials from the recordings (N = 14 trials) of dog behaviour and another independent naïve observer scored 25% (N = 17 trials) of owner behaviour. Reliability was excellent for all measures, see details in Table 2.

**Table 2:** Summarizes the coding measures and the definitions.

Measure	Definition	Location	Reliability (N = 14)
Approach	Whether dogs approaches owner in phase 1 (180 s) or phase 2 (360 s) or not at all	Target room	Unambiguous
Latency (in s)	Latency from the moment when the door closed as E leaves the first room until dogs enters target room by putting the first paw on one tile of the room	All rooms	Pearson Correlation r = 0.99
Direct approach	Dog approaches the target room immediately without a detour in less than 15 s	All rooms	Cohen`s Kappa = 0.70
Sniff (frequency and duration)	Dog is sniffing by making a sniffing noise, mouth closed in first room (before meeting owner) and in target room	First room; Target room	Pearson correlation r = 0.98 (first room frequency), r = 0.99 (first room duration), r = 0.99 (target room frequency), r = 1.00 (target room duration)
Sniff door (frequency and duration)	Dog is sniffing at the door close to the chair of the target person by making a sniffing noise, mouth closed	Target room	Pearson correlation r = 1.00 (frequency), r = 0.99 (duration)
Away from target room (frequency and duration)	After approach dog leaves target room; duration from dog being with four paws in second room until dog with all four paws back in target room	Second and first room	Pearson correlation r = 0.98 (frequency), r = 0.97 (duration)
Dog contact to owner (frequency and duration)	Dog in body contact with target person, clearly initiated by dog	Target room	Pearson correlation r = 0.99 (frequency), r = 0.96 (duration)
Lay down (occurrence)	Dog lays or sits down before approach (first room or entrance) or after approach (target room)	First room, entrance; Target room	Cohen`s Kappa = 0.63 (first room, entrance) Cohen`s Kappa = 1.00 (target room)
Behind target person (frequency and duration)	Dog approaches area behind the chair of the target person by putting one paw over the three tiles behind the chair	Target room	Pearson correlation r = 0.99 (frequency), r = 0.99 (duration)
Jump (frequency and duration)	Dog looks up or jumps up on the closets of the target room	Target room	Pearson correlation r = 1.00 (frequency), r = 0.99 (duration)
Sound (occurrence)	Dog makes a clear sound with his mouth, i.e. barking, whimpering, whining	All rooms	Cohen`s Kappa = 0.85
Owner contact to dog (frequency and duration)	Owner touches dog with his/her hand clearly initiated by owner	Target room	Pearson correlation r = 0.91 (frequency), r = 0.99 (duration)
Owner talk (frequency)	Number of utterances of the owner after the first greeting	Target room	Pearson correlation r = 0.94 (frequency)
Mean temperature of the day	Measured by: http://www.wetterkontor.de/de/wetter/deutschland/ (Wetterstation Jena Sternwarte)		
Rain (occurrence)	Measured by: http://www.wetterkontor.de/de/wetter/deutschland/ (Wetterstation Jena Sternwarte)		

## Results

Before testing the difference between experimental conditions, we asked whether any of the observed covariates has an effect on the dog approaching its owner in the first phase (which is a necessary condition for testing the expected effect). We evaluated a mixed effects logistic regression model with phase 1 approach as response (N = 101, 81 in which they do approach), and gender, age, number of trial, mean temperature, rain and education as fixed effects and dog identity as random effect. Only temperature (beta =  − 0.11, *p* = 0.007) appears to be associated with the response, so that higher temperatures make less probable for the dog to approach the owner.

For the following analysis we considered only those instances where the dog approached the owner. This amounts to N = 98 data points spread across 53 different dogs. Since some of the behavioral variables were missing for some of the observations, we imputed those missing entries using a random forest-based strategy with an OOB proportion of falsely classified observations equal to 0.11.

We first study dog’s behaviour before entering the room aiming at determining whether there are differential signs of excitement between the conditions (which is not to be expected since before entering the room the conditions should be equivalent). Since our hypothesis does not concern any individual behavioral trait, we project a number of variables into principal components. These variables are: the frequency and the duration of sniffing events in the first room, whether they approach the owner and if they do whether they do it directly, latency to approach owner and whether they lay down in the first room or entrance area. The first principal component explains 40% of the variation in the data and the signs of the projections are all such that the component can be regarded as reflecting more or less activity/excitement. We use this component (labelled as B1) as a summary of the behavior before entering the room. We regress B1 against condition and we add a number of covariates (age, gender, trial number, mean temperature, rain, education, owner greeting and group order) plus a random effect for dog identity. As expected, condition turns out not to be significant at conventional levels (0.32, *p* = 0.19) and only trial turns out to display a sizable and significant effect (− 0.62, *p* = 0.015), indicating that dogs show less activity in subsequent visits to the laboratory.

Upon entering the room we produce a similar behavioral summary as the one that yield B1, although in this case the variables considered are the frequency and duration of dogs contact to the owner, the frequency and duration of sniffing in the target room, the frequency and duration of leaving the target room, the frequency and duration of being behind the target person and jumping in the target room and whether they made a noise.

Once more, the first component explains a sizable proportion of the variation in the data (30%) and the signs of the projections let us interpret it as a general index of behavioral excitement (which we label B2).

Finally, we produce a PCA summary of the behaviour of the owner when greeting the dog. While it was stipulated that the owner should greet the dog “naturally”, there were some differences that might have influenced B2. We obtain the first principal component out of a set of three variables: Owner contact to dog (frequency & duration) and Owner talk (frequency), see Table 2. This main component (labelled “Owner greeting”) explains over 57% of the variance and the signs of the projection align with the notion of more/less activity from the part of the owner when greeting their dog.

**Figure 2:**
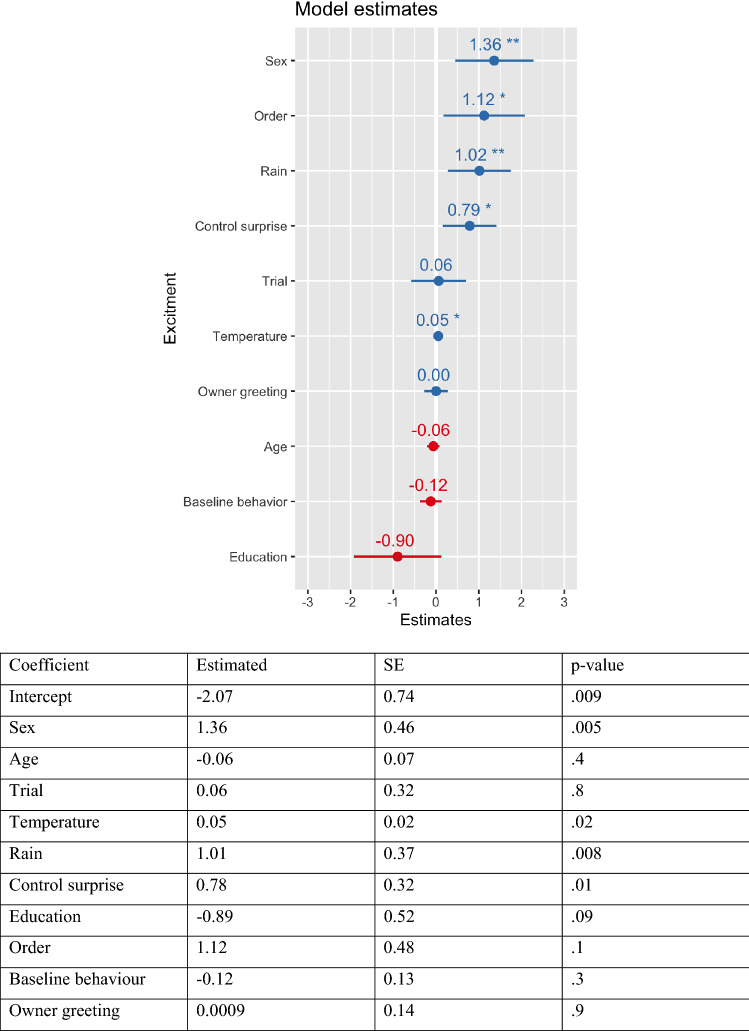
Estimated coefficients for the main model with behavioural excitement (B2) as response. Positive (blue) and negative (red) estimates are associated with increased and decreased excitement in the behavioural experiment. **p < 0.01; *p < .05.

We are now in position of testing the main hypothesis, namely that B2 differs substantially between experimental conditions. We regress B2 against condition, B1, Owner greeting, and the same set of covariates as before, see Fig. 2. Differences between the surprise and control conditions turn out to be positive and significant (0.79, *p* = 0.015), along with sex (1.29 for females, *p* = 0.006), mean temperature (0.05, *p* = 0.02), presence of rain (0.98, *p* = 0.01) and group order (1.12, *p* = 0.016). While these results reveal a clear behavioral difference in the two conditions and dogs do show more behavioral excitement in the surprise condition, it is important to note that significant covariates listed above have an effect that is comparable or higher than the main effect we evaluate here, and the same is true for the estimate standard deviation of the random effect for dog identity (SD = 1.05).

## Discussion

Dogs showed more behavioral excitement when the target person was replaced by another equally familiar person compared to the control condition in which the target person corresponded with the odour of the track. In other words, dogs` expectations were violated in the surprise condition. These results support the notion that dogs might hold a representation of their owners which includes information about their individual odor. Adding to the results of^33^ in which dogs searched for target objects, this is evidence that dogs represent what or who exactly they smell, i.e. that they do not perceive an odour as a relevant (positive or negative) or non-relevant stimulus, but that they really “match it with” what they are searching for.

The specifics of the olfactory information remain elusive. It has been recently conjectured that dogs might be able to trace through olfaction molecules of DNA^45^. However, this assumption has been called into question, since DNA pieces which are large enough for individual identification are too large to be sufficiently volatile to find their way into the nose of a sniffing dog^46,47^. But undoubtedly dogs and other animals are able to identify individuals by scent, although the exact process is not fully understood^45–48^.

Moreover, it was criticized in the study of Woidtke et al. (2018)^45^ that the trail was only 5 min old and the target person was present, so that the dogs could use air scenting. In the present study the time delay between laying the trail and searching was also rather short (15 min), and the target person was present—so that dogs could also use air scenting. Nevertheless, it is surprising that 16 out of 54 dogs did not approach the owner in the first 3 min (before they were called). On the other hand, another 16 dogs approached the owner immediately without any hesitation. This illustrates the huge inter-individual differences that cannot be explained by experience alone, as some trained rescue dogs also did not approach the owner in the first phase. We could, however, not detect a direct relationship between sniffing behavior and the latency of response in our main model—probably due to huge inter-individual differences. But it is possible that the dogs that approached the owner without any hesitation had sniffed already while they were in the entrance area before the trial started, so that this sniffing behavior was not coded.

In general, these findings support the assumption that dogs are not able to retrieve useful olfactory information unless they sniff^49^. In their neuroscientific review about dog olfaction, Gadbois & Reeve (2014) distinguish between “smelling” as being involuntary and implicit processed, and sniffing behavior that is exploratory and explicit processed. They describe that sniffing (i) actively participates in the input of the olfactory stimulus, (ii) can be modulated to account for different odorant concentrations, and (iii) can modulate the pattern of neural activity^50^. During sniffing, air is inhaled through the nostrils in short aspirations while the mouth is closed^51,52^. In that way odorant-laden air is drawn into the nasal cavity so that odorant molecules are transported from the external environment to olfactory receptor neurons in the sensory region of the nose^53^. Thus, sniffing creates a turbulent gas flow in the air passage and thus reduces the diffusion distance from fresh air to receptors in the nose^50,51^. There is an ongoing discussion whether dogs can pant and sniff simultaneously^54–56^, but it is plausible that sniffing is advantageous for odour perception compared to normal breathing.

Furthermore, we found that dogs were more efficient in finding the owner when the mean temperature was lower. This is in accordance with physiological studies suggesting that temperatures above 15 C reduce the physical capacity of dogs in motion because of hyperthermia^37,38^. In our study the target person was always present and air scenting was possible. Thus, it is conceivable that dogs` general decrease in searching performance when the temperature is high is more a consequence of physiological restrictions than environmental conditions that have impact on the source of the scent ^[see also 56]^. Thus, together with these anatomical and physiological findings, our results point out that dogs are not very sensitive in odour perception per se, but they have to sniff in order to find their owner who sits about 18 m away.

Interestingly, females showed substantially more behavioural excitement than males. Either they are more active in general or more motivated to find the owner. The latter seems more plausible as there are indications that females are more likely to interact with humans and better focus on single social stimuli^57^. Females outperform males when they are tested in the unsolvable problem task in which the subject initially learns to solve an easy task, which in the next phase of the test becomes impossible to solve, thus forcing the subject to pursue the objective alone or ask for help from human counterparts (see also above). Females then react with more social interactions with the experimenter^58,59^. Regarding olfactory skills, no sex differences are known, except that females might be less able to discriminate kin without the prerequisite of familiarity^60^ and males tend to sniff more odours in the sexual context^59^. Thus, the higher behavioral activity of the females in our study might be due to their higher sociability, which increases their motivation to find the owner.

We also found an effect for group order, indicating that it makes a difference for the dogs whether they are confronted with the surprise condition in their first or their second day of testing. In the study of Bräuer & Belger (2018), there was a different order effect: subjects showed “surprise”, i.e. hesitation to fetch the toy only in the first trial^33^. However, in that experiment all four trials were conducted in 1 day so that dogs could have perceived the smell of previous trials. In our experiment there was at least a week between the two trials so that it is highly unlikely that there was an interference with the odour of the previous trial. Indeed, we did not find an effect of trial number. Thus, dogs showed no significant difference in their behaviour between the first and the second day of testing as for example showing less (or more) activity in the second than in the first day because they were less motivated. There activity was mainly influenced by whether the target person was replaced or not.

However, in contrast to the study of Bräuer & Belger (2018), in which owners did not know the condition^33^, we could not prevent target persons from knowing whether they were replaced or not. They were not provided with details about the experimental setup or hypotheses, however it is still possible that they behaved differently depending on the condition, which justified the exclusion of all trials in which target persons did not only greet their dogs but held, fed or commended the dog. More importantly we analysed whether the way owners greeted their dogs by touching and talking had an influence on the behaviour of the dogs, and found no effect. It is however still possible, that dogs were influenced by different intensities of emotional cues provided by the owners while talking, as dogs are very sensitive to ostensive cues^61^.

In sum, or results bolster the notion that dogs develop a representation of the tracked target that is partially olfactory in nature. This leads to a number of questions of how odour perception and cognition are linked together, including the role of dog breed^7,48,62–64^ and how dogs decide to rely in olfactory or some other modality given the choice^54^. Our results lead naturally to interesting hypotheses about whether domesticated dogs have undergone a switch in their reliance of different modalities when comes to constructing cognitive representations. Finally, our findings might also shed light on the more general question how macrosmatic animals perceive the world through their nose.
